# Supplementary material for: Regional Variation of Chemical Characteristics in Young Marselan (Vitis vinifera L.) Red Wines from Five Regions of China
Source: Foods. 2022 Mar 9;11(6):787. doi: 10.3390/foods11060787 (PMC8948681; doi:10.3390/foods11060787)
Supplement: Supplementary file 1 [file foods-11-00787-s001.zip › foods-1610220-supplementary.pdf]

Supplementary material

Table S1. Number of Marselan wine samples with various vintages from different wine regions in China.

Wine region*	2012	2013	2014	2015	2016	Total
Jiaodong Peninsula		1	2	3	3	9
Bohai Bay				2	2	4
Huaizhuo Basin	1	1	1	4	3	10
Loess Plateau	1	1	1	1	3	7
Xinjiang			3	3	2	8

* Jiaodong Peninsula wines were collected from Qingdao, Yantai, and Weihai sub-regions. Bohai Bay wines were collected from Changli sub-region. Huaizhuo Basin wines were collected from Huailai sub-region. Loess Plateau wines were collected from Taigu and Linfen sub-regions. Xinjiang wines were collected from Manasi and Yanqi sub-regions.

Table S2. Climatic type of wine-producing regions in China in this study according to the Köppen-Geiger climate classification system.

Region	Sub-region	Climate type
Jiaodong Peninsula	Yantai	Monsoon-influenced humid subtropical climate (CWA)
	Qingdao	Monsoon-influenced humid subtropical climate (CWA)
	Weihai	Monsoon-influenced humid subtropical climate (CWA)
Bohai Bay	Changli	Monsoon-influenced hot-summer humid continental climate (DWA)
Huaizhuo Basin	Huailai[a]	Cold semi-arid climate (BSK)
Loess Plateau	Taigu	Cold semi-arid climate (BSK)
	Linfen	Cold semi-arid climate (BSK)
Xinjiang	Manasi[b]	Cold semi-arid climate (BSK)
	Yanqi	Cold desert climate (BWK)

[a] The climate type of Huailai region is based on Zhangjiakou City; [b] The climate type of Manasi region is based on Changji State.

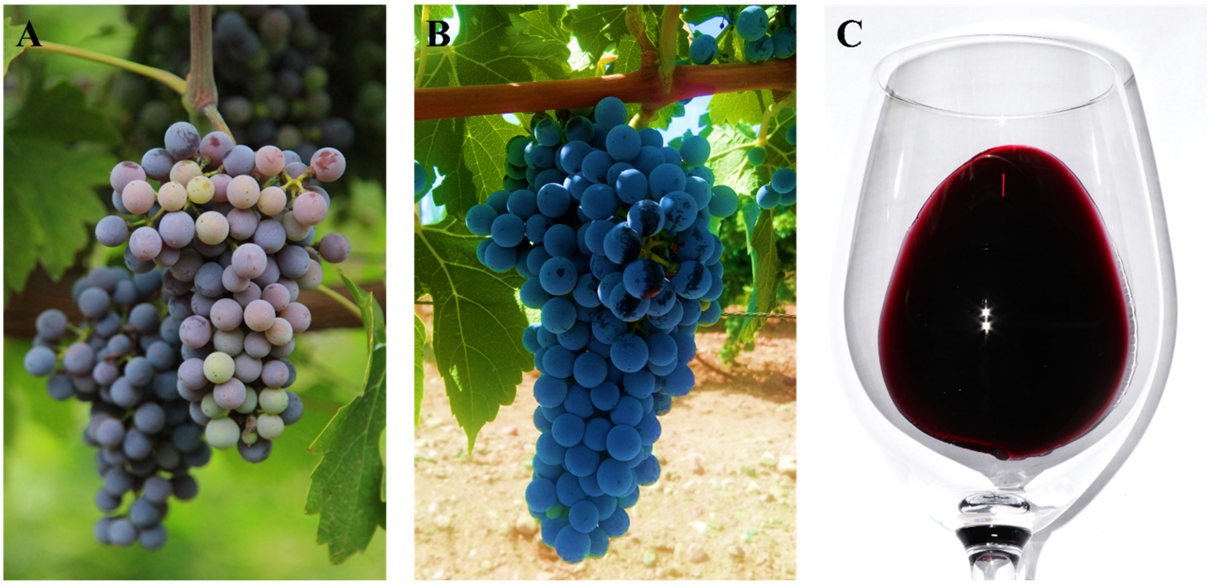

Figure S1. Photographs of Marselan grape at veraison stage (A) and mature stage (B), and young wine in a glass (C).

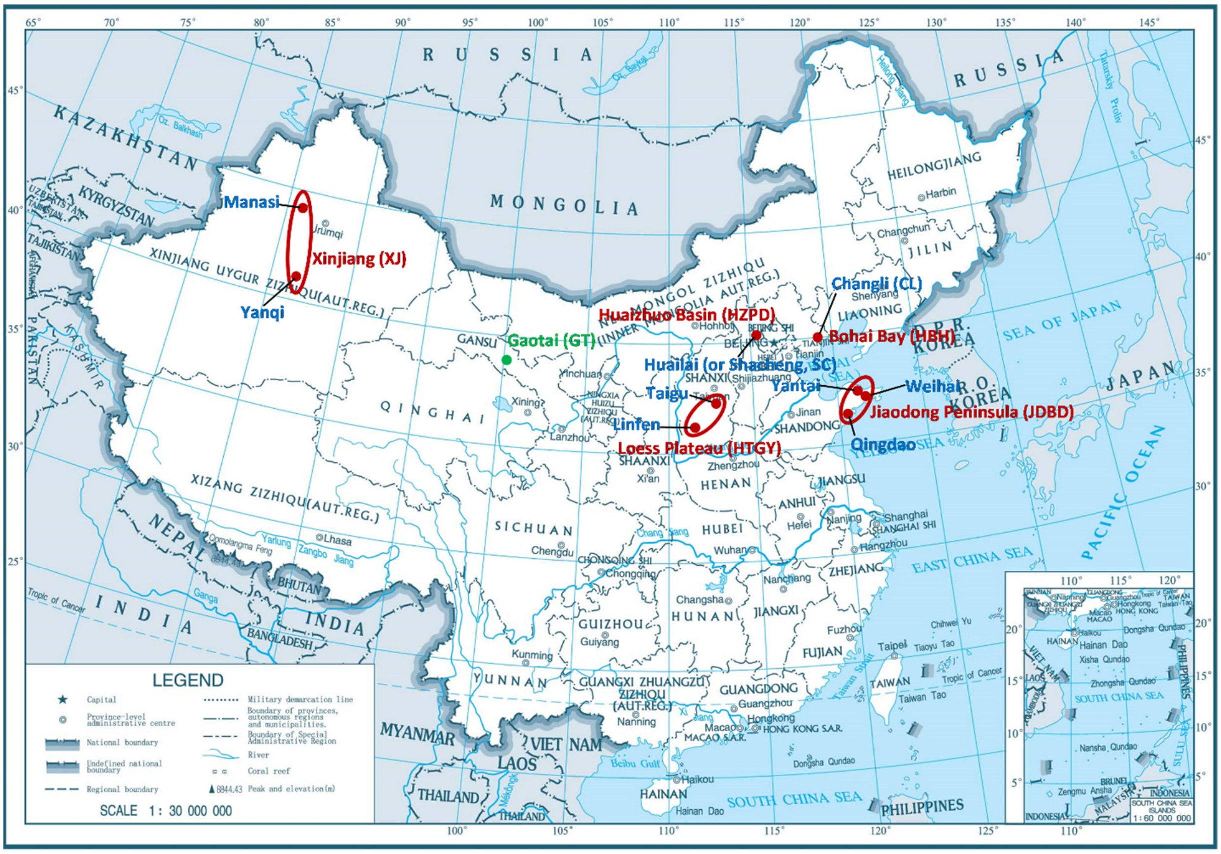

Figure S2. Regional distribution of wine production in China in this study.
